# Towards a Model of Contemporary Parenting: The Parenting Behaviours and Dimensions Questionnaire

**DOI:** 10.1371/journal.pone.0114179

**Published:** 2015-06-04

**Authors:** Carly A. Y. Reid, Lynne D. Roberts, Clare M. Roberts, Jan P. Piek

**Affiliations:** School of Psychology & Speech Pathology, Curtin University, Perth, Western Australia, Australia; University of Western Brittany, FRANCE

## Abstract

The assessment of parenting has been problematic due to theoretical disagreement, concerns over generalisability, and problems with the psychometric properties of current parenting measures. The aim of this study was to develop a comprehensive, psychometrically sound self-report parenting measure for use with parents of preadolescent children, and to use this empirical scale development process to identify the core dimensions of contemporary parenting behaviour. Following item generation and parent review, 846 parents completed an online survey comprising 116 parenting items. Exploratory and confirmatory factor analyses supported a six factor parenting model, comprising Emotional Warmth, Punitive Discipline, Anxious Intrusiveness, Autonomy Support, Permissive Discipline and Democratic Discipline. This measure will allow for the comprehensive and consistent assessment of parenting in future research and practice.

## Introduction

It has long been recognised that child development is affected by multiple interacting contextual factors at individual, familial, and societal levels [1]. One factor within this context that has been consistently identified as a significant predictor of child outcomes is parenting. There is a large body of research that suggests that parenting impacts on a range of domains in childhood, including academic, psychological, behavioural, and social [2–4]. In particular, various parenting dimensions, including psychological control, lack of parental warmth and autonomy support, and ineffective behavioural management, have been found to be strongly associated with internalising, externalising, and social problems in children in cross-sectional and longitudinal studies [5–7].

Factors such as evolution, family dynamics, culture, and social and political institutions have influenced parenting beliefs and practices over time [8]. However, contemporary parents and children are increasingly exposed to advertising, media, and new technologies, and parents may seek child rearing advice or information from a number of available sources, including websites, chat rooms, magazines, and television shows which offer a wide range of contradictory opinions about parenting. As a result, there is increasing ambiguity surrounding the behaviours that define “good” parenting, the acceptability of corporal punishment and other traditional disciplinary strategies, and expected behavioural and developmental standards for children [9–11].

In addition, there are several specific social trends that have occurred in recent decades that have had a significant influence on contemporary parenting behaviour. Since the 1970s, the number of women in the workforce has increased without a corresponding decrease in the number of men working, general material prosperity has increased, divorce rates have increased, and there have been significant changes in the structure and composition of the average family [12]. In addition, it appears that contemporary parents are spending more time working to provide material necessities for their children and less time interacting with children and addressing their emotional needs as compared to parents of previous generations [10].

Although children appear to be better off in many ways than children were 30 years ago, a number of adjustment and psychological problems, such as childhood depression and anxiety, are increasing in prevalence with the age of onset decreasing [13]. Furthermore, Loeber and colleagues [14] reported that externalising symptoms are the primary reason for referral to child and adolescent mental health services. These disorders are considerably disabling, and place a significant burden on individuals, families, and society as a whole with regards to both direct and indirect costs [13], [15]. As parenting has been found to have a significant impact on the developmental trajectory of both internalising and externalising problems [16], [17], it appears that there is a pressing need for the comprehensive and accurate definition and measurement of key parenting behaviours that both contribute to and protect against childhood maladjustment outcomes.

While a substantial amount of theoretical and empirical research over the past six decades has focused on the concept of parenting, there is a lack of agreement regarding the key elements and assessment of this construct. A large proportion of the literature has been based on Baumrind’s [18–20] parenting styles. However, much of this research uses a simplified two factor typology of emotional warmth and behavioural control to distinguish four parenting styles. This does not take into account the complexity of Baumrind’s original descriptions, including elements such as democracy and autonomy granting. In addition, there has been an increasing focus on the dimension of psychological control in research on adolescent outcomes, a factor not traditionally associated with Baumrind’s typology, and there is evidence that it may be important to examine this dimension in the parenting of preadolescent children as well [16], [21], [22], [23]. The small number of studies that have included the dimension of psychological control in the parenting of preadolescent children have indicated that it has specific and unique effects on a number of important childhood outcomes, including internalising and externalising symptomology [16], [23].

It has also been argued that important information may be lost in combining parenting dimensions into styles, and disaggregating these typologies will allow the individual key elements of parenting to be operationally defined, assessed, and combined into meaningful styles or patterns [24], [25]. Researchers will then be able to examine the independent, cumulative, and interactive effects of these dimensions on childhood outcomes, and the dimensions will provide the foundations for comprehensive and comparable parenting assessment in future research and clinical practice [25].

O’Connor [26] and Skinner et al. [25] agreed that the parenting dimensions identified in any current model may not adequately describe the phenomenology of parenting, and that there may be other dimensions that warrant further attention. Indeed, a number of parenting constructs have been proposed in the literature, including democracy [27], involvement [28], other-oriented discipline [29–32], monitoring [33], corporal punishment [34], contingent discipline [35], and inconsistency [36]. In addition, constructs such as anxious intrusiveness [37], power assertion and love withdrawal [29–32], behavioural, emotional, and cognitive psychological control [38], conditional regard [39], hostility [40], dependency-oriented and achievement-oriented psychological control [41], overprotection and overindulgence [42], scaffolding [43], [44], responsiveness [45], and invalidation, guilt induction, excessive expectations, ridiculing, embarrassing in public, comparing to others, ignoring, and violation of privacy [46] have all been described as psychologically controlling or autonomy supportive parenting behaviour. However, it is unclear whether these dimensions can be subsumed under the themes of warmth, behavioural control, and psychological control, or whether they comprise distinct parenting dimensions that have unique effects on childhood outcomes. In addition, there does not appear to be any current parent self-report assessment that comprehensively assesses all of the relevant dimensions and subdimensions in parents of preadolescent children. It is possible that previously unidentified parenting dimensions may be better able to predict child adjustment outcomes, which highlights the importance of using rigorous empirical methodology to uncover the true underlying factor structure of a measure in order to identify and assess the salient practices of contemporary parents. It is clear from the literature that a comprehensive but economical, psychometrically sound, and high utility parenting measure is needed in order to advance research into the optimisation of parenting behaviour and childhood adjustment outcomes.

Therefore, this research aimed to develop an inclusive assessment of parenting through which the key elements of parenting could be identified and operationally defined, and the specific effects on childhood outcomes could be investigated. It was hoped that the comprehensive identification of important parenting elements would allow for clear comparison of parenting behaviour across parent, child, family, and cultural groups, and greater cohesion between the various theoretical conceptualisations of parenting, and provide an indication of the important parenting behaviours to assess in the development of future observational and interview assessment measures.

This research was based on a wide range of theoretical parenting conceptualisations, self-report assessments, and qualitative and quantitative input from a sample of parents. A mixed-method approach was employed in order to combine the expertise of parenting researchers with the endorsement of contemporary parents in selecting important and relevant items for inclusion in the final Parenting Behaviours and Dimensions Questionnaire (PBDQ).

## Study 1: Item Development

DeVellis [47] suggested that scale development begin with a review of the substantive theories of interest in order to establish a theoretical foundation for the scale, and items for inclusion in the initial item pool should be developed to reflect this theoretical basis as well as the purpose of the scale. However, there is a huge diversity of parenting dimensions that have been hypothesised and included in assessments developed by the experts in the parenting field over the past sixty years. This has meant that there is no unanimous agreement over a single, comprehensive theory of parenting or set of definitive, core dimensions that this measure could be based on [26]. Thus, although a comprehensive review of the parenting literature was conducted, including the theoretical conceptualisations of parenting and the development and psychometric evaluation of current parenting instruments, items that were generated for the item pool were based on pre-existing parenting measures that were selected due to their utility, availability, theoretical and psychometric support, and assessment of a range of parenting factors considered to be important by experts in the parenting field. According to DeVellis [47], expert review of the item pool is an important step in scale development, as experts can confirm or invalidate the relevance of items, and provide feedback on clarity, redundancies, item length, reading difficulty, and the use of ambiguous terminology, as well as highlight any relevant theoretical areas that have not been included. Experts are generally considered to be people who have worked extensively with the chosen construct [4]. However, 192 of the 210 items in the initial item pool were based on existing questionnaire items proposed by a number of experts within the parenting field, rather than being written by the researcher (see Table 1), and can be considered to represent a range of expert opinions. As a result, the experts consulted in the current project to assess content validity were a sample of parents. Parents were consulted to ensure that the items that were originally developed by the experts in the field were also considered clear, valid, and practically relevant in assessing the parenting behaviour of contemporary parents. Some items were also added according to suggestions made by parents that were consistent with parenting literature and agreed upon by the research team.

**Table 1:** Parenting Measures Included in the Initial Item Pool.

Measure	Author	Scope	Subscales	Items
PS	Arnold et al., 1993	Dysfunctional discipline styles	Laxness, overrreactivity, verbosity	30
APQ	Shelton et al., 1996	Practices related to externalising problems	Parental involvement, poor monitoring/supervision, inconsistent discipline, positive parenting, corporal punishment, other discipline practices	42
PSDQ	Robinson et al., 1995	Baumrind's parenting typology	Authoritative parenting (warmth and involvement, reasoning/induction, democratic participation, good natured/easygoing); authoritarian parenting (verbal hostility, corporal punishment, non-reasoning/punitive strategies, directiveness); permissiveness (lack of follow through, ignoring misbehavior, self-confidence)	62
PAQ-R	Reitman et al., 2002	Baumrind's parenting typology	Authoritative, authoritarian, permissive parenting	30
PCRQ	Furman & Adler, 1983	Qualities of the parent-child relationship	Warmth, personal relationship, disciplinary warmth, power assertion, and possessiveness	57
WPI	Weinberger, Feldman, & Ford, 1989	Parent attitudes and behaviours	Child-centredness power assertive discipline, inconsistent/permissive parenting	49

*Note*. PS = Parenting Scale; APQ = Alabama Parenting Questionnaire, PSDQ = Parenting Styles and Dimensions Questionnaire, PAQ-R = Parental Authority Questionnaire- Revised, PCRQ = Parent-Child Relationship Questionnaire, WPI = Weinberger Parenting Inventory.

### Method

This study was approved by the Curtin University Human Research Ethics Committee. In study 1, participants provided written consent to participate. They were provided with an information and consent form, which they were invited to send back in a separate provided envelope to ensure confidentiality.

#### Participants

Sixteen parents recruited from advertisements throughout the researcher’s university, community newspapers, and snowballing participated in this study. Participants were all mothers, with ages ranging from 31 to 51 years (*M* = 41.71, *SD* = 7.48). Number of children per family ranged from one to four, with children’s ages ranging from 3 to 17 years of age; however all parents had at least one child aged between 3 and 12 years.

A further 15 parents of children aged 3 to 12 years of age were recruited for focus groups using community advertisements and snowballing. Participants were all female with ages ranging from 32 to 51 years (*M* = 39.64, *SD* = 5.57). Number of children per family ranged from one to five, with children’s ages ranging from 2 to 18 years of age.

#### Measures

The 270 items of six parent self-report measures were selected as the initial item pool: the Parenting Scale [48], Alabama Parenting Questionnaire [49], Parenting Styles and Dimensions Questionnaire [50], Parenting Authority Questionnaire- Revised [51], Parent Child Relationship Questionnaire [52], and Weinberger Parenting Inventory [53]. These measures were chosen as they assessed a range of important parenting styles and dimensions, were parent self-report rather than child-reported or retrospectively reported, were freely available, validated for use with parents of preadolescent children, and included the most frequently used measures in the literature. A list of 18 items related to dimensions of autonomy support, intrusiveness, and overprotection was also generated by the research team due to the underrepresentation of these dimensions in the included questionnaires. The combined measures yielded a total of 288 items. Upon review by the research team, 90 items unanimously deemed redundant were eliminated, 10 double-barrelled items were reworded into two separate items and 1 triple-barrelled item reworded into three separate items. This resulted in an initial item pool of 210 items.

It was decided that the response format of the new measure would focus on the frequency of parenting behaviours (a 5-point Likert-type scale response format ranging from 1 (*never*), 2 (*sometimes*), 3 (*about half the time*), 4 (*often*), and 5 (*always*) rather than assessing attitudes or beliefs, as behavioural frequency appears to be more reliably reported by parents in previous research [54].

A child age range of 3 to 12 years was chosen for the measure, as Roberts et al. [55] reported relative stability in parenting practices used with children in this age range.

#### Procedure

Permission was received from authors of the original measures to include them in this research. The 16 parents initially recruited were asked to review the parenting questions in the initial item pool and make suggestions, cross out, or write comments about the questions (participants did not complete the questionnaire). This included questions that didn’t make sense, were ambiguous, unclear, or difficult to answer, were badly worded, repetitive, irrelevant or inappropriate, or any other comment. The order that the lists were presented in was alternated, with the option to provide feedback on only one list if preferred. Parents were then asked to return the comments on the questionnaires to the researcher in the reply paid envelope.

Three focus groups were conducted using the 15 additional parents recruited, with six participants in focus group one, five participants in focus group two, and four participants in focus group three. At the commencement of the focus group, parents were given a brief outline of the importance of parenting and the purpose of the current research. Participants were then asked some general questions about their perspectives on parenting, including what defined them as a parent, what were the most important things about the way that they parented, what they thought made a good parent, and what things they wanted to avoid as a parent. Participants were then asked to read through the parenting questions, and discuss whether the questions were relevant, made sense, were clearly worded, or repetitive, and finally, if there were any questions or themes that they felt were missing from the list. Focus groups ran for an average of 156 minutes.

### Results

The initial 16 participants provided written feedback on 210 items, with 16 items receiving no comments from parents, suggesting that they were unanimously deemed clear and appropriate. Written comments for each question were collated, and suggestions for additional items were listed separately. Tapes from the three focus groups were transcribed. Specific item feedback was extracted from each transcriptand combined with the individual parent comments. Content analysis [56] was then conducted on each transcript with a focus on manifest meaning. The categories or themes chosen for the current analysis were reflective of specific parenting behaviours that had not been assessed by the items in the item pool, as well as any general feedback on the item pool that was not item specific. Twenty one themes and accompanying quotes were combined with suggestions for additional items provided by participants. As a result, 21 items were added to the item pool, using original participant quotes to aid in the wording of the additional items.

**Fig 1:**
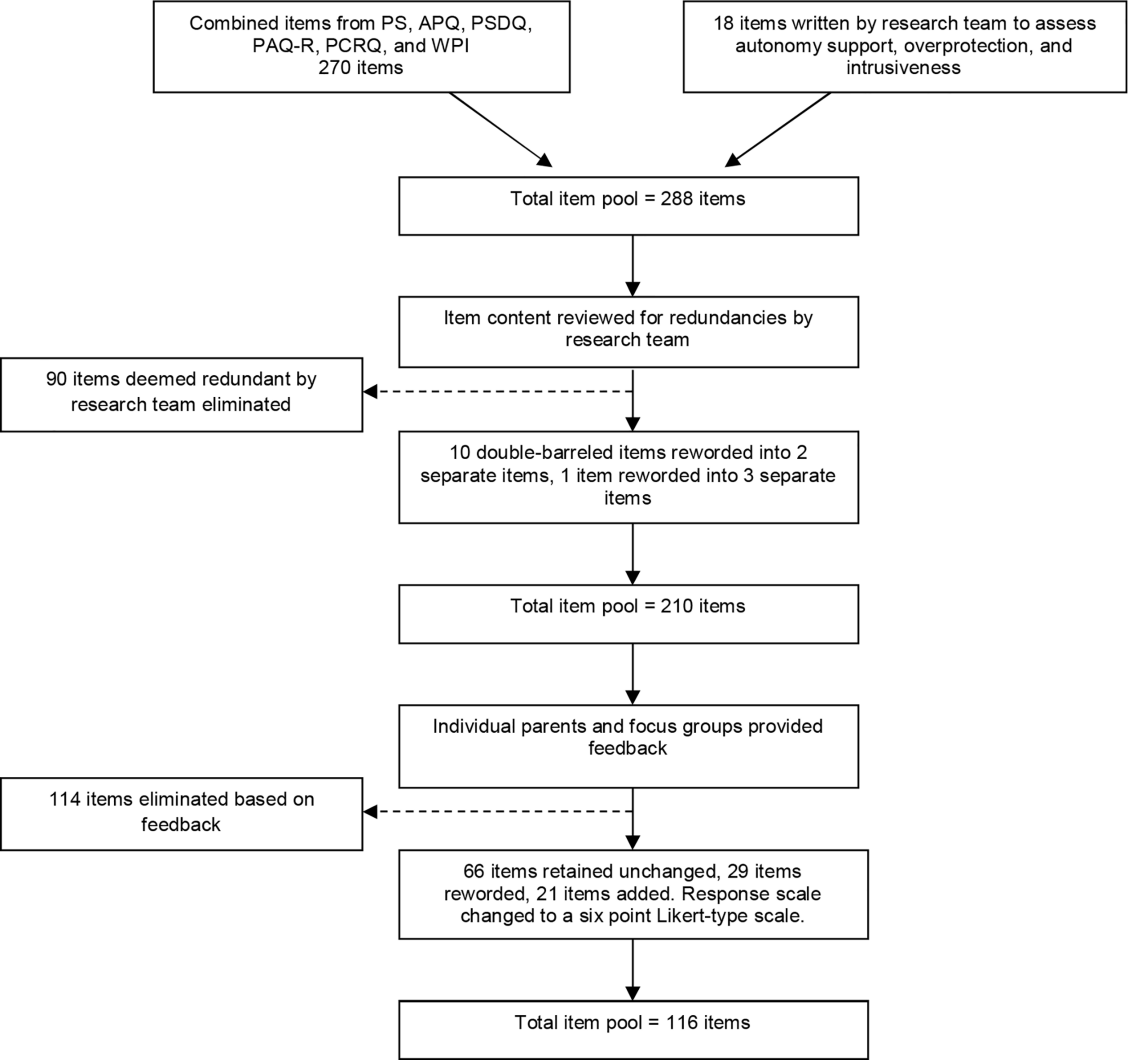
Summary of item selection process.

Each item with comments was evaluated by the research team, comprising a PhD (Clinical Psychology) student, a professor of developmental psychology, and an associate professor and clinical psychologist with extensive experience in parent research, training, and assessment, and child behaviour therapy. Two members of the research team are parents. Each item was evaluated based on the number of comments made by participants, similarity of concerns raised, and agreement amongst the research team concerning the validity of the feedback raised in relation to the parenting literature. One hundred and fourteen items that were redundant, difficult to answer, or inappropriate were deleted. Items that were badly worded, double-barrelled, or unclear were reworded where appropriate. Items that were not unanimously voted by the research team to be deleted were retained. In total, sixty six items were retained unchanged, 114 items were eliminated, and 30 items were reworded, with two of these items reworded into one item. A ‘*rarely*’ option was also added to the Likert scale, as suggested by individual and focus group participants. Final response options therefore included 1 (*never*), 2 (*rarely*), 3 (*sometimes*), 4 (*about half the time*), 5 (*often*), and 6 (*always*). The final list of questions contained 116 items. A summary of the item selection process is presented in a flow chart presented in Fig 1.

### Discussion

The aims of Study One were to develop an initial item pool for the proposed PBDQ that reflected the expertise of parenting researchers, and employ a sample of contemporary parents to conduct an expert review of the items. Following these processes, a total of 116 items were included in the final item pool. The response scale for the proposed PBDQ was changed from a five point Likert-type scale to a six point Likert-type scale due to the participants’ suggestion that a ‘rarely’ response option be included.

A diverse range of parenting issues and behaviours were considered relevant and important by parents in the current study. Not all of these issues have been identified by previous parenting measures, but many of these appear to be consistent with concepts discussed by previous parenting researchers. Other added items did not appear to be directly related to parenting literature, but are discussed in other areas of psychological literature. For example, the modelling of behaviour, values, and attitudes relates back to constructivist theories [57], while encouraging or rewarding good behaviour is discussed in operant conditioning [58], and praising effort rather than focusing on achievement has been researched in relation to motivational styles [59].

The combination of written parent comments and focus group data in the current research combined the advantages of both methodologies. Giving parents the opportunity to provide written feedback allowed them complete the questionnaire at a convenient time and place, and the feedback was individual and therefore less likely to be influenced or coerced by others [60]. In addition, the methodology was less intrusive and more private than interviews or focus groups [61], which may have been beneficial in discussing sensitive parenting issues such as the use of corporal punishment. On the other hand, Kitzinger [62] suggested that the group discussion of focus groups is particularly advantageous when the researcher intends to encourage participants to explore and discuss issues that are important to them using their own vocabulary, which was the case in the current study. Krueger and Casey [63] explained that focus group participants may also take their cues from other participants' comments, and therefore greater exploration of a range of perceptions can be achieved due to a process of ongoing and dynamic activation of the participants’ relevant memories or thoughts.

The combination of a number of different sources of expertise was important as there are a large number of parenting conceptualisations in the literature and a lack of agreement over the core dimensions to assess [26]. Finally, the items developed for this measure were designed to assess the frequency of parenting behaviours rather than assessing attitudes or beliefs, as previous measures of parenting have been criticised due to their inability to provide specific behavioural information, and behavioural frequency appears to be more reliably reported by parents [54]. Therefore, the current research combined the advantages of several methodologies, including literature review, consultation of previous assessments, individual parent feedback, and focus groups, in order to produce a reduced item pool of 116 items for the empirical development of the proposed PBDQ.

One limitation of the study was that no fathers responded to the advertisements to participate. It is possible that this sample bias may have impacted on the items that were retained in the initial PBDQ item pool.

## Study 2: Factor Structure of the PBDQ

The aim of Study 2 was to determine the factor structure underlying the parenting items for the proposed PBDQ using a combination of Exploratory Factor Analysis (EFA) and Confirmatory Factor Analysis (CFA).

### Method

This study was approved by the Curtin University Human Research Ethics Committee. In study 2, an online questionnaire was used. The link provided in advertising the survey directed participants to an information sheet housed on the Curtin University website outlining the purpose of the study, the rights of the participants, and the procedure. Participants were informed that clicking on the link indicated that they gave consent to participate in the study. Participants were instructed to complete the demographics questionnaire, followed by the parenting questionnaire items. Once the parenting questionnaire items were completed, participants were directed to a debriefing page housed on the Curtin University website.

#### Participants

Participants comprised a convenience sample of 846 parents of at least one child aged 3 to 12 years, recruited by advertising through radio, community newspapers, online parenting and research forums, and snowball sampling. Participants included 763 females and 77 males (gender data missing for six participants) and were aged between 19 and 57 years (*M* = 35.85, *SD* = 6.76). Participants were asked to select one of their children to answer the questionnaire items about, as there may be differences in the behaviours used by the same parent with different children in their family [64–66]. This was based on the parent’s decision, and no guidance was given about which child to select.

The majority of the parents who completed the questionnaire lived in Australia (77.42% of total sample), self-described their ethnic identity as Australian (65.84%), and were mothers (89.48%), and primary caregivers (86.05%). In addition, the majority of participants were highly educated, with 76.35% having commenced or completed a university qualification.

Almost 82% of participants had partners who lived in the home, and 69.57% of all partners were married. More than half of the partners (54.14%) had commenced or completed some university or obtained a university qualification. Participants indicated that 51.89% of their partners considered their ethnic identity to be Australian, and 18.44% had suffered from psychological problems at some point in their life.

A large majority of the sample (90.19%) came from families with three children or less, and almost 70% of these families were comprised of the child’s mother and father living together. The age of the child chosen by the parent ranged from 3 to 12 years (*M* = 6.85, *SD* = 2.84), and most of these were first born children (66.19%). Slightly more male children (50.83%) were chosen than female children (47.87%), and parents indicated that 7.68% of children had suffered from psychological problems at some point in their life.

#### Materials

The reduced list of 116 parenting items developed in the previous chapter was used for this study. These items asked participants to report how often they engaged in the behaviours specified in the items, rated on a 6 point Likert-type scale, ranging from 1 (*never*) to 6 (*always*).

A questionnaire assessing a number of parent, child, and family related demographic variables was used in this study.

#### Procedure

Demographic questions and the PBDQ item pool were formatted into an online survey using the limesurvey.com platform. Internet data collection was used in the current study as a cost-effective means of obtaining a large and diverse sample of parents as participants without affecting the nature and quality of the results [67]. In line with best practice [68], the survey was sandwiched between an information sheet and debriefing page hosted on a university website. Items from the PBDQ item pool were presented immediately following the demographic questions. The child’s age and parenting questionnaire items were set to forced response so that all of these items had to be answered. Upon completion of the survey participants were invited to enter a draw to win a $100 voucher, a strategy previously shown to increase survey response and retention rates [69].

#### Data Analysis

Data for the EFA were collected over a six month period. Cases (*N* = 815) were downloaded from limesurvey.com into an Excel file, which were then imported into a Predictive Analytics Software (PASW) database. Once the data were screened for non-valid responses (*N* = 778 cases remaining), 580 cases were randomly selected for the EFA using PASW.

Data were downloaded from limesurvey.com again five months later into an Excel file (*N* = 888) and imported into a PASW database. Once these data were screened, the additional 68 cases collected were combined with the 198 remaining cases from the previous PASW database for the CFA (*N* = 266).

### Results

#### Exploratory Factor Analysis

Assumption testing indicated the data were suitable for factor analysis, with the sample size of 580 meeting the minimum ratio of five cases per variable [70]. Principal Axis Factoring (PAF) with Promax rotation (Kappa = 4) was selected as the aim was to identify the latent variables that caused the manifest variables to covary [71]. Oblique rotation was chosen over orthogonal rotation, as the literature suggests that some factors, such as warmth and autonomy support, may be correlated [19], [20], [50].

Parallel analysis was conducted at the beginning of the analysis, and after the elimination of each item, to determine the number of factors to retain [72]. Initial parallel analysis indicated that ten factors should be retained, which was specified as the value of the fixed number of factors to extract in the initial PAF analysis. The minimum criteria for item retention was a primary factor loading greater than or equal to 0.32, and cross-loadings on a secondary or tertiary factor less than 0.32 as recommended by Tabachnick and Fidell [73]. Items were eliminated individually, beginning with those items with the lowest primary factor loadings. A total of 41 items were eliminated as they failed to meet the minimum primary factor loading criteria. Items cross-loading on a secondary or tertiary factor greater than 0.32 were then eliminated as recommended by Tabachnick and Fidell, beginning with those items with the highest cross loadings. Following the removal of each item, all remaining items were re-evaluated against the minimum primary loading criteria before being re-examined for significant cross-loadings. The elimination of significant cross-loadings resulted in the removal of a further 15 items.

A six factor solution was achieved, with all items meeting the minimum criteria for significant primary factor loading and non-significant cross-loadings. Further items were then removed with the aim of producing a brief measure that minimised burden on respondents while retaining acceptable reliability [47]. Twenty-two items were removed based on lack of theoretical fit and/or the impact on the reliability of the scales. One item was also removed for utility purposes, as it was the only remaining reverse-scored item in the questionnaire. Cronbach’s alpha remained above the acceptable level of 0.7 [74] for all scales.

The final EFA six factor solution consisted of 36 items. The pattern factor loading matrix for this solution is presented in Table 2. Factor loadings ranged from .39 to .80, with the majority above .50. Factors, labelled based on previous literature [20], [37], [49], [53], [75], [76], were Emotional Warmth, Punitive Discipline, Autonomy Support, Permissive Discipline, Anxious Intrusiveness, and Democratic Discipline.

**Table 2:** Final Pattern Factor Loading Matrix for EFA.

Item	Factor Loading					
	EW	PD	AI	AS	PerD	DD
I tell my child how proud I am of him/her	.73					
I respond to my child’s feelings or needs	.68					
I show an interest in my child’s life	.65					
I show my child that I love them unconditionally	.63					
I recognise my child’s strengths and talents	.56					
I make time for my child when he/she needs me	.50					
The punishments that I decide on are influenced by my mood		.75				
I lose my patience when my child does something to upset me		.75				
I punish my child more severely than I mean to		.70				
I am easy on my child one minute, and hard on him/her the next		.68				
I threaten my child with punishments that I would never actually use		.48				
I worry about my child when he/she is not at home			.61			
I am more concerned with my child’s feelings than my own			.55			
I share more of my life with my child than with anyone else			.54			
I try to anticipate what my child’s desires are and provide them before he/she has to ask			.52			
I rely on my child to cheer me up when I’m feeling down			.50			
I try to shield my child from experiencing negative emotion			.46			
I try to meet my child’s desires immediately			.46			
I encourage my child to try things for him/herself before asking for help				.74		
I encourage my child to problem solve				.62		
I let my child try to figure things out for him/herself before giving my input				.61		
I adjust my level of assistance in tasks based on my child’s age and ability				.44		
I encourage my child to choose his/her own interests and activities				.43		
I give my child responsibilities appropriate to his/her age				.42		
I don’t punish my child when he/she has misbehaved					.59	
I allow my child to interrupt other adults					.54	
I do things for my child when he/she refuses to do them					.54	
I ignore my child’s misbehaviours					.49	
I don’t punish my child if he/she acts sorry					.45	
I give in to my child when he/she gets upset					.42	
I do things for my child that he/she is capable of doing for him/herself					.39	
I talk to my child about the consequences of his/her actions						.80
I explain to my child how I feel about his/her behaviour						.68
I let my child know what behaviour is expected						.51
I give my child reasons about why he/she isn’t allowed to do something						.51
I encourage my child to consider the consequences of their choices before making them						.43

*Note*. EW = Emotional Warmth, PD = Punitive Discipline, AI = Anxious Intrusiveness, AS = Autonomy Support, PerD = Permissive Discipline, DD = Democratic Discipline.

#### Confirmatory Factor Analysis

CFA was conducted on the separate, independent sample of 266 participants. Two models were compared: a correlated six factor model, and an uncorrelated six factor model. Following testing of assumptions, CFA was conducted using EQS version 6.1, utilising the robust statistics option. The Lagrange Multiplier (LM) test indicated three items had significant cross-loadings with other factors and were removed from the analysis. These items were ‘I rely on my child to cheer me up when I’m feeling down’, ‘I encourage my child to problem solve’, and ‘I ignore my child’s misbehaviours’. Four fit indices; the Comparative Fit Index (CFI), the Non-Normed Fit Index (NNFI), the Root Mean Square Error of Approximation (RMSEA) and the Satorra-Bentler chi-square divided by degrees of freedom; were used to evaluate model fit. The fit statistics for the 33 item correlated and uncorrelated models are summarised in Table 3. The correlated model demonstrated acceptable fit statistics, meeting the minimum value for all robust fit estimates. Table 4 shows the final pattern factor matrix for the CFA.

**Table 3:** Comparison of Correlated and Uncorrelated CFA Models.

Model	S-B χ^2^	S-B χ^2^/df	CFI	NNFI	RMSEA	RMSEA upper limit	RMSEA lower limit
*Cut-off Criteria*		< 2	= / >.85	= / >.85	= / <.06	<0.08	close to 0
Original Correlated Model	952.19	1.65	0.86	0.85	0.05	0.06	0.04
Correlated Model with 3 Items Removed	675.8	1.51	0.9	0.89	0.04	0.05	0.04
Uncorrelated Model	1157.34	2.34	0.73	0.71	0.07	0.08	0.07

*Note*. S-B χ^2^ = Satorra-Bentler Chi-square statistic, S-Bχ^2^ /df = Satorra-Bentler chi-square divided by degrees of freedom, CFI = Comparative Fit Index, RMSEA = Root Mean Square Error of Approximation.

**Table 4:** Final Pattern Factor Loading Matrix for CFA.

Item	Factor Loading					
	EW	PD	AI	AS	PerD	DD
I tell my child how proud I am of him/her	.67					
I show an interest in my child’s life	.70					
I show my child that I love them unconditionally	.71					
I make time for my child when he/she needs me	.74					
I respond to my child’s feelings or needs	.73					
I recognise my child’s strengths and talents	.64					
I lose my patience when my child does something to upset me		.55				
I threaten my child with punishments that I would never actually use		.60				
I am easy on my child one minute, and hard on him/her the next		.76				
The punishments that I decide on are influenced by my mood		.74				
I punish my child more severely than I mean to		.61				
I worry about my child when he/she is not at home			.24			
I am more concerned with my child’s feelings than my own			.40			
I share more of my life with my child than with anyone else			.43			
I try to anticipate what my child’s desires are and provide them before he/she has to ask			.68			
I try to shield my child from experiencing negative emotion			.49			
I try to meet my child’s desires immediately			.53			
I encourage my child to choose his/her own interests and activities				.44		
I encourage my child to try things for him/herself before asking for help				.56		
I adjust my level of assistance in tasks based on my child’s age and ability				.54		
I give my child responsibilities appropriate to his/her age				.71		
I let my child try to figure things out for him/herself before giving my input				.56		
I give in to my child when he/she gets upset					.65	
I don’t punish my child if he/she acts sorry					.39	
I do things for my child that he/she is capable of doing for him/herself					.44	
I do things for my child when he/she refuses to do them					.72	
I allow my child to interrupt other adults					.54	
I don’t punish my child when he/she misbehaves					.49	
I let my child know what behavior is expected						.64
I encourage my child to consider the consequences of their choices before making them						.56
I give my child reasons about why he/she isn’t allowed to do something						.44
I talk to my child about the consequences of his/her actions						.77
I explain to my child how I feel about his/her behavior						.70

Using the combined EFA and CFA dataset, mean scores were created for each of the six factors using the average score of the items which had their primary loadings on each factor, with higher scores indicating greater use of the parenting behaviours described by the dimension. A mean score was chosen due to the unequal number of items in each factor, allowing for greater ease of comparability between subscales. Sample descriptive statistics for the six factors are displayed in Table 5. Inspection of the distribution of the 33 items included in the final measure indicates a moderate degree of skewness (range of skewness -2.2 to 1.4, standard error .08) and kurtosis (range of kurtosis -1.27 to 6.3, standard error .17) of some items. These values fall below the cut-offs of 3 and 8 respectively that indicate data is unsuitable for analysis without transformation [77]. Visual inspection of the histograms and Q-Q plots suggested that all factors excepting Emotional Warmth and Punitive Discipline approximated normality. The Emotional Warmth factor appears to be significantly negatively skewed and leptokurtic, while the Punitive Discipline distribution appears to be moderately positively skewed. This is consistent with previous research, which shows that parents in community samples generally score highly in dimensions of warmth and acceptance, and low in harsh, punitive discipline [78–81]. Cronbach’s alpha for the Anxious Intrusiveness subscale was only minimally acceptable in the combined sample (.60 for subscales with less than 10 items [82]).

**Table 5:** Sample Descriptive Statistics for Final Solution (N = 846).

Factor	Score	α	*M*	*SD*	K-S *p*	Skewness		Kurtosis	
	Range					Raw score	*z*-score	Raw score	*z*-score
Emotional Warmth	2.67–6.00	0.83	5.53	0.46	0	-1.64	-19.48	4.81	28.63
Punitive Discipline	1.00–5.20	0.79	2.24	0.71	0	0.87	10.33	0.91	5.39
Anxious Intrusiveness	1.50–5.83	0.66	3.49	0.8	0	0.28	3.3	-0.3	1.8
Autonomy Support	2.60–6.00	0.7	5.06	0.54	0	-0.6	-7.15	0.89	5.32
Permissive Discipline	1.00–4.50	0.7	2.52	0.61	0	0.33	3.88	0.16	0.93
Democratic Discipline	3.00–6.00	0.75	5.2	0.56	0	-0.7	-8.27	0.46	2.76

*Note*. K-S = Kolmogorov—Smirnov.

Correlations between factor scores are displayed in Table 6. As expected, some medium to large significant correlations were found between composite scores, specifically between Emotional Warmth and Punitive Discipline, Anxious Intrusiveness, Autonomy Support, and Democratic Discipline; Punitive Discipline and Autonomy Support, Permissive Discipline, and Democratic Discipline; Autonomy Support with Permissive Discipline and Democratic Discipline; and Permissive Discipline with Democratic Discipline.

**Table 6:** Correlation Between Factors in Final Solution (N = 846).

	Emotional Warmth	Punitive Discipline	Anxious Intrusiveness	Autonomy Support	Permissive Discipline	Democratic Discipline
Emotional Warmth	1.00	-.37*	.26*	.49*	-.19*	.53*
Punitive Discipline		1.00	.12*	-.32*	.35*	-.31*
Anxious Intrusiveness			1.00	.01	.20*	.05
Autonomy Support				1.00	-.29*	.49*
Permissive Discipline					1.00	-.29*
Democratic Discipline						1.00

*Note*.

*p < .01.

### Discussion

This research aimed to use empirical procedures to produce a brief but comprehensive assessment of key contemporary parenting dimensions. Following item development and evaluation, EFA and CFA were used to support the development of a multidimensional measure of parenting, including Emotional Warmth, Punitive Discipline, Anxious Intrusiveness, Autonomy Support, Democratic Discipline, and Permissive Discipline. The resultant *Parenting Behaviours and Dimensions Questionnaire* (PBDQ) has a sound factor structure and good internal consistency for all subscales.

The Emotional Warmth factor, consistent with previous descriptions of warmth [20], [28], [78] reflects the degree of affection and emotional support that parents show toward their child, with higher scores indicating high levels of acceptance, display of positive affect, and receptiveness shown to the child. Half of the items were suggested by contemporary parents in the item development phase, supplemented by on researcher generated item, and two items from previous measures [50], [53].

Emotional warmth facilitates the development of a sense of competence, agency, and trust in the child, providing the foundation for confidence and competence in social interactions as well as high academic achievement [83], [84]. When emotional warmth is absent, a number of adverse outcomes may arise, including elevated levels of hostility, dependence or detachment, and anxiety, poor self-esteem and self-adequacy, emotional unresponsiveness and dysregulation, and a negative worldview [85–87].

Previous research suggests that punitive, power assertive, or physical punishment methods are associated with poor outcomes in children [34], [88]; and in the current study, Punitive Discipline emerged as the second strongest factor. All Punitive Discipline items were based on items included in the WPI [53] Higher scores on this subscale reflect higher levels of harsh, psychological, and mood-dependent discipline strategies. This Punitive Discipline factor appears to be consistent with authoritarian parental control which is forceful, punitive, and restrictive, as opposed to authoritative control, which is democratic, clearly explained, rational, and firm [18–20]. This factor also appears to reflect power assertive behaviours as described by Hoffman [29], [30], [89]. Parental rejection or hostility, reflected in this Punitive Discipline subscale, may be part of the psychological control dimension, including criticism, hostility, aggression, harshness, ignoring, and neglect [40], [46], [90]. The current findings support Grolnick and Pomerantz’s [91] proposal that authoritarian and psychologically controlling strategies, such as the use of force, intrusiveness, curbing initiative, power assertion, and failing to take the child’s perspective are separate from structure, referring to setting rules and limits, and provision of an organised and predictable environment [91], [92], as items describing the latter content did not load on this factor. This also reflects the distinction between behavioural control and autonomy support discussed by Barber [24].

The Anxious Intrusiveness factor was given the same name as the factor in Becker’s [37] research, which he described as tendencies toward infantilising and overprotection, and oversolicitousness for the child's safety and happiness. These behaviours were thought to discourage the development of autonomy and independence in children [37]. Two items from this list were developed by the researchers, one item was suggested by parents who participated in the focus groups, and other items were based on items from existing measures [50], [52], [53]. Higher scores on this subscale indicate higher levels of parental enmeshment, intrusive assistance, and indulgence.

Intrusive parental behaviours reflect unrealistic expectations of the child’s developmental level and their capabilities, and may involve infantilising behaviour and the provision of excessive and unnecessary assistance [93]. Many of these items also relate to parental overprotection [94], [95], which includes overpossessiveness, domineering behaviour, overgratification of the child’s wishes, and intrusive attempts to protect the child from experiencing disappointment and distress [96–99]. This reflects noncontingent, protective, and indulgent parenting strategies which are thought to impact on the child’s sense of mastery, agency, self-efficacy and perceived control, and prevent them from learning effective coping and emotional regulation skills [100–102]. In this sense, Anxious Intrusiveness appears to be related to psychological control as defined by SDT [103], [104], and particularly dependency-oriented psychological control, which refers to behaviours that encourage enmeshment and discourage individuation [41], [105]. This is often considered the opposite of responsive autonomy support [106], [43], [107]. However, a very low correlation was found between Anxious Intrusiveness and Autonomy Support (*r* = -.03) in the current study. It is important to note that Cronbach’s alpha for the Anxious Intrusiveness subscale was only minimally acceptable in the combined sample (.60 for subscales with less than 10 items) [82]. Further research is needed to investigate the reliability of this subscale across different sample groups.

The next factor to emerge was Autonomy Support, referring to scaffolding and responsive parenting behaviour. Low scores may reflect developmentally inappropriate, intrusive, and unsolicited assistance [108] and restrictive control or forcing the parent’s own agenda [20], [91], [109], [110]. Such behaviour may occur as a result of parental anxiety or unrealistic expectations of their child’s abilities [107]. Large correlations between Autonomy Support and Emotional Warmth (*r* = .45), and Democratic Discipline (*r* = .48) indicate that high parental Autonomy Support is also associated with higher Democracy and Emotional Warmth, consistent with theorised relationships [111, 112].

Two of the items in the Autonomy Support factor were suggested by parents in focus groups, with the remaining three items originated from the researchers. This dimension has not been adequately assessed in earlier parenting measures [23], [16], [21] Self-report measures of autonomy support generally ask parents to rate how often they provide directions or assistance, which does not include consideration of the needs and abilities of the child [106]. The child’s age, needs, and abilities are explicitly referred to in two of the Autonomy Support items, which is a significant strength of this PBDQ subscale.

Permissive or inconsistent discipline has long been associated with externalising problems in children [113], as well as the development of an external locus of control [114–116]. Permissive Discipline reflects the setting and enforcing of rules and expectations. One of the Permissive Discipline items was generated by the researchers, while the remaining items were based on items from existing questionnaires [48], [49], [50]. Permissive Discipline items appear to describe laissez-faire parents, who allow their children a great degree of behavioural freedom even if their actions affect others in a negative way [96]. This factor also includes failure to follow through with demands and promises, erratic changes in expectations and behavioural consequences, indiscriminate responses to the child’s behaviours, and giving in to the child after initially resisting Permissive Discipline appears to be consistent with chaos, which is the opposite of firm behavioural control or structure [18], [19], [20], [91]. Some items in the Permissive Discipline factor appear to be related to a form of psychological control, as these behaviours are not responsive to the developmental needs of the child. The immediate alleviation of a child’s distress is not conducive to optimal development of independent regulation [117] and noncontingent parenting behaviour may undermine the child’s sense of agency, autonomy, and competence, and result in the child developing an unpredictability schema about their environment [114]. Permissive Discipline is negatively associated with Autonomy Support (*r* = -.28), which reflects contingent, responsive parenting behaviour.

The final factor to emerge from the EFA was labelled Democratic Discipline, which reflected the parent’s use of reasoning and explanation. One of these items was suggested by parents in the focus groups, while the remaining items were based on items from previous questionnaires [50], [51], [52]. Democratic Discipline is associated with authoritative parenting [18–20] and is the main distinguishing factor between firm control and restrictive, superfluous control used by authoritarian parents [118]. Previous research suggests parents who use democratic discipline are high in loving acceptance and autonomy [76], provide structure in an autonomy supportive way [109], and are responsive to the needs of the child [110]. Reflecting this, Democratic Discipline was highly correlated with Emotional Warmth (*r* = .51) and Autonomy Support (*r* = .48), and negatively correlated with the noncontingent disciplinary factors of Punitive Discipline (*r* = -.30) and Permissive Discipline (*r* = -.29).

It appears that the PBDQ dimensions of Emotional Warmth, Autonomy Support, and Democratic Discipline are associated with autonomy supportive parenting behaviour which is administered in a responsive and contingent manner, while Punitive Discipline, Anxious Intrusiveness, and Permissive Discipline describe non-contingent and unresponsive parenting behaviours that can be described as psychologically controlling. These results provide strong support for the importance of assessing autonomy supportive and psychologically controlling behaviours in parents of preadolescent children.

Interestingly, most of the dimensions uncovered in this research appear to relate to the conceptualisations proposed by Baumrind [18–20] and to a lesser extent, Hoffman [29], [30], [119]. Although not distinguished as separate dimensions, Emotional Warmth and Democratic Discipline are consistent with descriptions of Baumrind’s [18–20] authoritative parenting style. In addition, authoritative parenting was later described in terms of high levels of responsiveness and demandingness by Maccoby and Martin [120] and Baumrind [121], [122] and these are consistent with low levels of Permissive Discipline and high levels of Autonomy Support respectively. Behaviours associated with authoritarian parenting include punitive, forceful, and autonomy restrictive behaviours and discouragement of democracy and explanation [18], which appears to be consistent with high levels of Punitive Discipline and low levels of Autonomy Support and Democratic Discipline in the current conceptualisation. Authoritarian parenting is also generally thought to be characterised by low levels of Emotional Warmth and Permissive Discipline. Baumrind [123] also explained that authoritarian parents may be protective and concerned, which could relate to Anxious Intrusiveness. Permissive parents were described by Baumrind [18–20] as non-punitive and democratic, but they do not attempt to shape or assist in the regulation of the child’s behaviour. This parenting style appears to be most strongly related to Permissive Discipline, but these parents may also score highly on Emotional Warmth and Democratic Discipline, and low on Autonomy Support. These parents are also unlikely to engage in Punitive Discipline, although Baumrind stated that permissive parents may also be indulgent and protective, which is consistent with Anxious Intrusiveness. The final parenting style of neglectful parenting, which was later added by Maccoby and Martin [120] does not appear to be consistent with any particular dimensions of the PBDQ; however, we can hypothesise that involved parenting would correspond to low scores on all of the PBDQ dimensions. These results suggest that the PBDQ dimensions may represent a disaggregation of Baumrind’s parenting styles into composite dimensions, which will allow for the examination of their unique, relative, and combined contributions to childhood outcomes.

In addition, Autonomy Support and Democratic Discipline appear to reflect Hoffman’s [29], [30], [119] concepts of other-oriented and inductive discipline, while power assertion and Punitive Discipline also seem to describe similar parenting behaviours. However, love withdrawal, which involves ignoring the child, rejection, and expression of anger [29], [30], [119], does not appear to be closely related to any of the PBDQ dimensions. Interestingly, Lewis [118] suggested that the provision of responsive, democratic, and inductive parenting is more important than parental control in promoting positive child outcomes. Indeed, it seems that Autonomy Support and Democratic Discipline can be broadly described as autonomy supportive parenting behaviours as they are administered in a responsive manner, with Emotional Warmth also thought to be a feature of responsive parenting [106], [112]. On the other hand, Punitive Discipline, Permissive Discipline, and Anxious Intrusiveness describe unresponsive parenting behaviours that undermine the child’s autonomy, and are therefore considered psychologically controlling. It therefore appears that an important component of all of the PBDQ dimensions is the way in which the behaviour is administered, and this is more important in describing contemporary parenting practices than the use of specific control strategies.

Indeed, one of the major contributions of the PBDQ is the assessment of parental autonomy support or responsiveness in parents of preadolescent children. This dimension appears to be related to the administration of specific parenting behaviours, such as assistance, behavioural control, and comfort, in a way that takes into account the child’s developmental level, needs, and abilities. Johnston and colleagues [106] explained that self-report measures that ask how often parents assist their child in tasks or provide directions do not accurately capture this dimension; however, the Autonomy Support items included in the PBDQ specifically ask parents about the child’s age, needs, and abilities, and discuss the encouragement of initiative and problem solving before asking for help. Interestingly, all of the Autonomy Support items were derived from suggestions made by participants or the list of researcher generated items related to responsiveness and autonomy support, suggesting that existing parenting measures have not adequately assessed autonomy supportive, responsive parenting behaviour. Studies on the parenting of preadolescent children have rarely included a measure of autonomy support or psychological control, although features of these dimensions are sometimes assessed as part of the parental warmth or behavioural control dimensions [16].

Although the original item pool for the PBDQ included items from assessments of parenting styles, homogenous parenting dimensions emerged in the factor analysis. Furthermore, these dimensions had differential relationships with other PBDQ dimensions. For example, Emotional Warmth was moderately correlated with Anxious Intrusiveness, while the related concept of Autonomy Support did not appear to be related to this dimension at all. These results provide support for the assessment of disaggregated parenting dimensions rather than focusing on aggregated parenting styles.

The PBDQ can be used to improve comprehensiveness, quality, consistency, and accuracy of parenting assessment in both research and clinical settings. The dimensions identified by the PBDQ can also provide the foundations for the development of alternative and more complex parenting assessment systems, including observational measures, research and clinical interviews, and child-report measures. These can then be used to collect comprehensive and valid multitrait multimethod parenting assessment data that addresses problems such as shared method variance. Grolnick [109] suggested that previous studies may have underestimated the magnitude of relationships between parenting and child outcomes due to shared method variance and unreliable or inconsistent methods, and therefore the use of more complex assessment systems based on core dimensions may result in larger and more clinically and practically significant results.

The evaluation of such parenting interventions or clinical studies could also be achieved by administering the PBDQ before and after the intervention is conducted to determine if any meaningful changes in parenting behaviour have occurred. This measure has the additional advantage of being relatively brief and economical, which minimises the burden of participant responses and increases the utility of the measure, particularly for research purposes [47]. As a result, it could also be used for large scale screening of children or families at risk for poor psychosocial outcomes, allowing for early identification, targeted intervention, and identification of clinical samples for future parenting research. Further research establishing the concurrent and predictive validity of the PBDQ will also allow researchers and clinicians to predict the influence of parenting dimensions on academic, social, and psychological outcomes in children. The PBDQ could also be administered longitudinally to determine which parenting dimensions are stable over time and which may be amenable to intervention.

A major strength of the PBDQ is the rigorous methodology that was used in developing the scale, combining previous parenting literature and existing parenting assessments with qualitative parent feedback and empirical assessment of the item and overall model performance. The combination of a number of different sources of expertise was particularly important as the content of the questionnaire was not mapped onto domains of interest as a result of the large number of parenting conceptualisations in the literature and the lack of agreement over the core dimensions to assess [26]. In addition, mixed-method designs provide the study with the combined advantages of qualitative and quantitative research, and allow the researcher to integrate and draw conclusions using the different perspectives that are gained through these methodologies [124].

One limitation of the current study was the failure to use random sampling in asking parents to choose a child to answer the parenting items about. This may represent a source of bias in the responses; for example, parents may have chosen the child who they employed the most socially desirable parenting behaviours with. It is also possible that the higher education level attained by the participants in all phases of this research as compared to Australia’s national average as reported by the Australian Bureau of Statistics [125] may have impacted on the items that were retained in the initial PBDQ item pool, as well as the resultant factor structure. Furthermore, generalisability of results may be affected by the self-selection process of participation. Parents who volunteered to participate may be more interested, committed, and involved in parenting, more willing to change their behaviours to become more effective parents, and more open to information about parenting, than other parents who did not choose to participate. Finally, the majority of the parents who completed the questionnaire lived in Australia, and were mothers and primary caregivers. Future research is needed to replicate the factor structure and psychometric properties of the PBDQ across larger and more diverse samples of parents, including low socioeconomic groups, ethnic minority groups, non-primary caregivers, and groups of fathers, where random sampling procedures are used.

Although a diverse range of parenting issues and behaviours were considered relevant and important by parents in Phase One of this research, many of these items failed to meet the minimum loading criteria on any factor in the EFA. It is possible that the decision to interpret the suggestions made by parents as indicative of specific parenting behaviours rather than broader parenting constructs may have contributed to this outcome. Indeed, further factors may have emerged in the EFA if more items had been developed to reflect these broader themes, which could be an area of future development of the PBDQ model. In addition, the decision to use a Likert scale anchored by ‘Never’ and ‘Always’ may elicit trait judgments in respondents and it is possible that some of the assessed content may vary over time and situations.

## Conclusion

The newly developed PBDQ is a brief but comprehensive measure of contemporary parenting behaviour that measures six core dimensions of parenting: Emotional Warmth, Punitive Discipline, Anxious Intrusiveness, Autonomy Support, Permissive Discipline, and Democratic Discipline. These dimensions combined a number of different concepts from previous literature, providing some clarity to the definition of key parenting dimensions as well as highlighting the similarities and differences between a number of parenting concepts that vary in terminology, definition, theoretical basis, and assessment. The measure was developed using rigorous scale development procedures, and the results of this study provide preliminary support for factorial validity and internal consistency.
